# Pectoralis Major Tear with Retracted Tendon: How to Fill the Gap? Reconstruction with Hamstring Autograft and Fixation with an Interference Screw

**DOI:** 10.1155/2017/2095407

**Published:** 2017-01-30

**Authors:** L. Baverel, K. Messedi, G. Piétu, V. Crenn, F. Gouin

**Affiliations:** ^1^CHU de Nantes, Clinique Chirurgicale Orthopédique et Traumatologique, Hôtel-Dieu, Place A. Ricordeau, 44093 Nantes Cedex, France; ^2^LPRO, Inserm UI957, Laboratoire de la Résorption Osseuse et des Tumeurs Osseuses Primitives, Faculté de Médecine, Université de Nantes, 44000 Nantes, France

## Abstract

Rupture of the pectoralis major tendon is considered an uncommon injury and a significant number of ruptures are missed or diagnosed late, leading to a chronic tear. We report an open reconstruction technique and its outcomes in a case of chronic and retracted PM tear. At the last follow-up (12 months), the patient was pain-free, with a visual analogic scale at 0 all the time. He was very satisfied concerning the cosmetic and clinical results. The constant score was 93%, the SST value 95%, and the Quick DASH score 4.5. MRI performed one year postoperatively confirmed the continuity between PM tendon and graft, even if the aspect of the distal tendon seemed to be thinner than normal PM tendon. The excellent clinical outcomes at one-year follow-up suggest that PM tear with major tendon retraction can be reliably reconstructed with hamstring autograft, using a bioabsorbable screw to optimize the fixation device. This technique has proven its simplicity and efficiency to fill the gap.

## 1. Introduction

Rupture of the pectoralis major (PM) tendon is considered an uncommon injury occurring in male patients between 20 and 40, most being of military population and athletes [1, 2]. The incidence seems to increase with both weight lifting practice and use of anabolic steroids [3]. Nonspecific clinical signs are ecchymosis and pain, but more specific is a loss or thinning of the anterior axillary fold [4]. Magnetic resonance imaging (MRI) is the gold standard to confirm diagnosis, localize and grade the tear, and measure the stump retraction and the muscle fatty degeneration [5]. Surgical repair during the acute phase is recommended, regarding excellent outcomes and low number of operative complications [6–12].

Pectoralis Major is well described as a two-head muscle, according to its clavicular and sternocostal heads [13]. Its humeral tendon insertion is just lateral to the bicipital groove and measures approximately 5 centimeters in length and 3 to 4 millimeters in width, with U-shape (anterior and posterior layers inferiorly continuous) [14]. According to Bak, complete tears are more common than partial tears, with, respectively, reported rates of 91% and 9%. However, significant number of PM injuries are missed or diagnosed late, leading to a chronic tear [4, 15, 16]. Some authors reported good clinical outcomes after direct sutures of chronic PM tears, once tendon was released and mobilized [12].

Otherwise, tendon graft is necessary in presence of chronic tear with significant tendon retraction and altered tissue quality [17]. Various graft techniques have been described: hamstrings autograft [16], bone-patellar bone-tendon autograft [18], fascia lata allograft [19], Achilles tendon allograft [4], and dermal allograft [15]. In the literature, numerous fixation devices have been reported and compared, as suture anchor [4], unicortical button [20, 21], bone trough [12], or transosseous suture [6]. Authors found no significant biomechanical difference between these fixation devices [22–24]. However, interference screw seems to be equal or superior to theses other modes of fixation for subpectoral tenodesis of the long head of the biceps [25–29].

We report an open reconstruction technique and its outcomes in a case of chronic and retracted PM tendon tear. The tendon reconstruction was performed with hamstrings autograft fixed with a humeral interference screw. To the best of our knowledge, this technique has not been reported in the literature.

## 2. Case Report

A 30-year-old male, street-cleaner-worker, sustained a right (dominant) shoulder injury in a motorcycle accident. He was heavy manual worker and did not practice any sport. In the emergency department, an acromioclavicular joint dislocation was initially diagnosed, and the patient was treated in a conservative manner. One year later he presented to the senior author (LB) with complaints of pectoral pain and cramps and deformity of the chest. He had significant functional limitations; mainly return to work was impossible. Physical exam revealed an abnormal anterior axillary contour and reduced adduction and internal rotation strength. The shoulder range of motion was however complete. The constant shoulder score was 51 [30], the simple shoulder test 30% [31], and the quick DASH score 52.3 [32].

**Figure 1:**
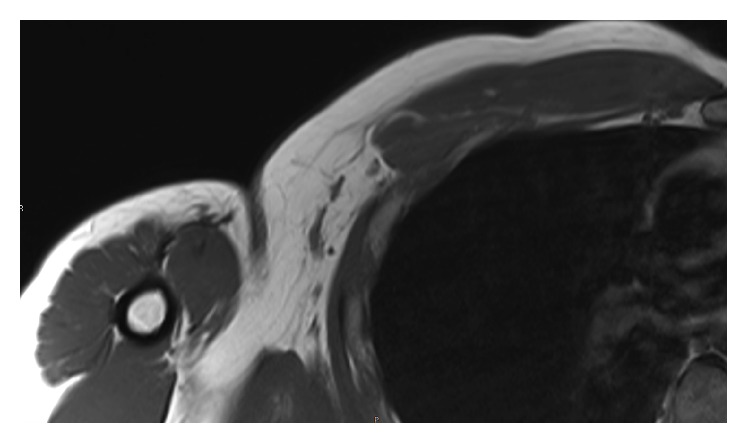
MRI axial T1 showing full-thickness PM tear at the humeral tendon-bone junction.

**Figure 2:**
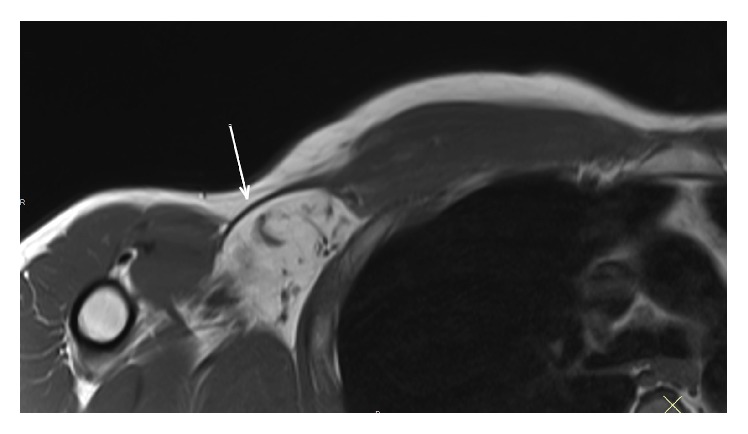
MRI axial T1 showing tendon retracted medial to the anterior chest wall and absence of any muscle fatty infiltration.

**Figure 3:**
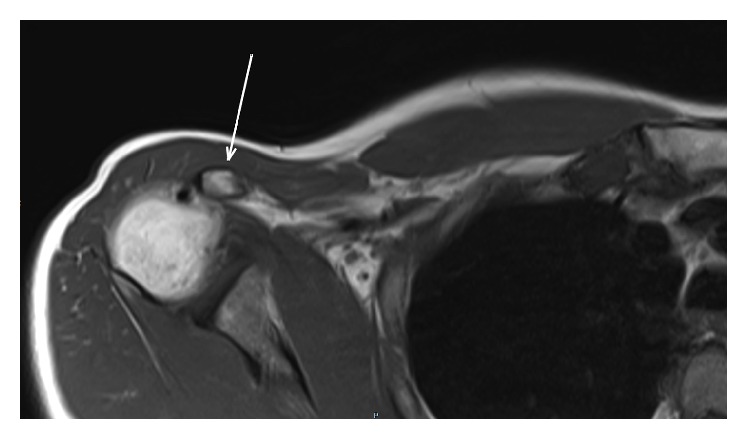
MRI axial T1 showing calcification inside the conjoint tendon immediately under its coracoid insertion.

Standard shoulder X-ray did not reveal any abnormality. MRI identified (1) full-thickness PM tear at the humeral tendon-bone junction including both pectoral heads (Figure 1), (2) tendon retracted medial to the anterior chest wall (Figure 2), (3) absence of any muscle fatty infiltration, and (4) a calcification inside the conjoint tendon immediately under its coracoid insertion (Figure 3). The lesion corresponded to C2/F/C after ElMaraghy and Devereaux [33]. Surgical management was considered regarding major daily activity impairment. The patient consented to surgical procedure once detailed explications were given about PM repair with autograft hamstring tendon to fill the gap.

## 3. Operative Technique

**Figure 4:**
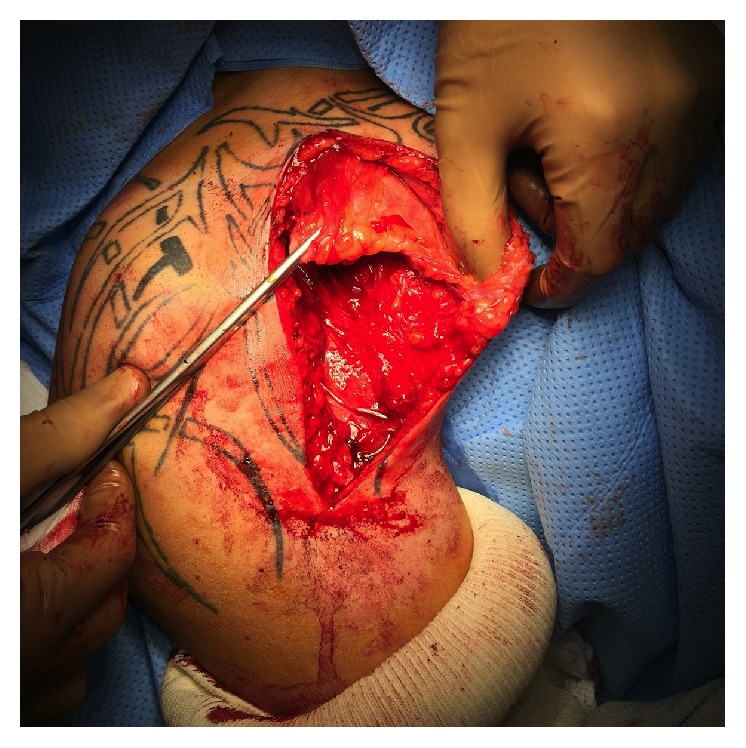
Intraoperative photograph demonstrating the gap between the footprint and the stump.

An interscalene block was performed before surgery, and the patient was operated under general anaesthesia in the beach-chair position. Ipsilateral knee was positioned in 90° flexion with an air tourniquet applied to the limb and draped free. A deltopectoral approach was first performed. The proximal part of the incision was more medial than the standard approach to ease pectoral muscle release. The distal part of the incision was enlarged to the PM footprint. The operative findings confirmed both the full-thickness PM tendon tear and the retraction of the tendon that was positioned more medial than the anterior chest wall. Despite extensive muscle release, the tendon could not be approximated to its anatomic insertion. The gap between the footprint and the stump was more than 5 cm (Figure 4).

**Figure 5:**
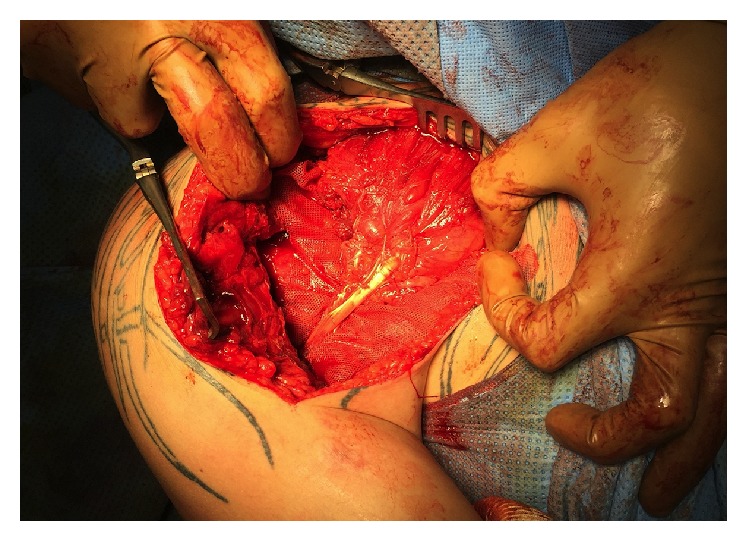
Intraoperative photograph, with the free border of the graft sutured at the PM.

**Figure 6:**
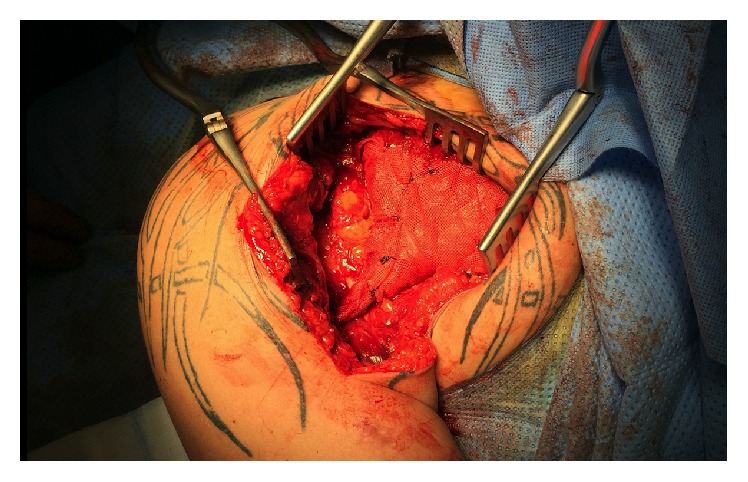
Intraoperative photograph with Vicryl plate wrapped around the graft.

Tendon reconstruction with hamstrings was confirmed. Semitendinous and Gracilis tendon were harvested through an oblique anteromedial approach, [34] using a tendon stripper (Smith & Nephew). The tendons were cleaned of soft tissue and folded to form 7 cm length 6 strands. It was stitched along its distal part to obtain a fan-shape tendon. The diameter at the distal part of the graft was calibrated at 9 mm. The lateral aspect of the bicipital groove was exposed, while the biceps tendon was carefully protected. The humeral tunnel was performed at the PM center footprint. The humeral tunnel was matched size for size with the graft diameter and had a depth of 25 mm to be bicortical. Two centimeters of hamstring graft was fixed within the bone tunnel with a 9 mm *∗* 25 mm bioabsorbable screw (Biosure, Smith & Nephew). The fan-shaped free border of the graft was sutured into the muscle belly with Mason-Allen and Krachow stitches using nonabsorbable suture (Ultrabraid, Smith & Nephew) arm in neutral rotation (Figure 5). A Vicryl plate (Ethicon) was wrapped around the graft, in order to secure the sutures (Figure 6). The calcification in the conjoint tendon was removed.

## 4. Postoperative Care and Rehabilitation

The arm was immobilized postoperatively in a sling for 6 weeks. Passive closed chain pendulum exercises were initiated immediately after the surgical procedure, until 45° of abduction during 21 days and 90° for the three weeks later. No external rotation was allowed for six weeks. Active range of motion, stretching exercises, and external rotation were then initiated. Dynamic strengthening was delayed past three months, once complete range of motion was obtained. Return to heavily activities at work was allowed after 6 months.

## 5. Results

**Figure 7:**
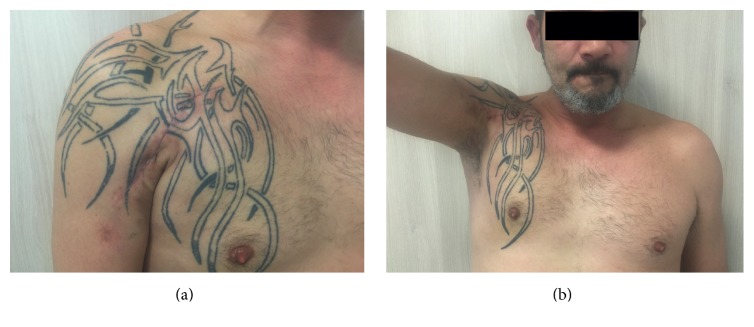
At two months postoperatively, the anterior axillary contour was restored (a), and the range of motion was 110° in lateral elevation (b).

The drain was removed 2 days after surgery and then the patient was discharged. There was no early complication regarding the PM reconstruction and no morbidity at the donor site. At two months postoperatively, the anterior axillary contour was restored (Figure 7(a)), and the shoulder range of motion was 130° in anterior elevation, 110° in lateral elevation, 10° in external rotation, and 5° in internal rotation at 90° of abduction (Figure 7(b)). X-ray confirmed the correct position of the screw and the absence of osteolysis around it. Six months after surgical reconstruction, the patient was pain-free. The axillary anatomy was restituted and shoulder range of motion was complete. Therefore, return to work was authorized.

**Figure 8:**
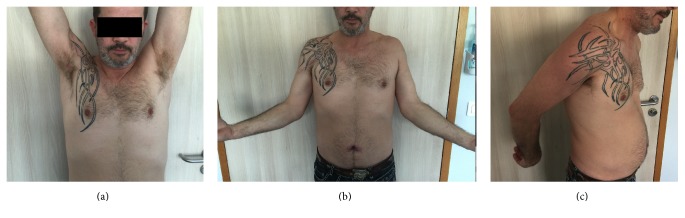
Complete range of motion in elevation (a) and in external rotation (b) and negative lift-off test (c).

At the last follow-up (12 months), the patient was pain-free, with a visual analogic scale at 0 all the time. He was very satisfied concerning the cosmetic and clinical results (Figures 8(a)–8(c)). The constant score was 93%, the SST value 95%, and the Quick DASH score 4.5. After Bak's criteria [7], the patient was classified as excellent: no symptoms, normal range of motion, no cosmetic modifications, no adduction weakness, and work without restriction. MRI performed one year postoperatively confirmed the continuity between PM tendon and graft, even if the aspect of the distal tendon seemed to be thinner than normal PM tendon.

## 6. Discussion

To our knowledge, this is the first description of a full-thickness PM tendon tear with gap, successfully filled with hamstring autograft fixed with interference screw. PM tears are uncommon and can be easily missed during initial presentation, leading to delayed diagnosis and treatment [4, 15, 16]. In a meta-analysis of 112 cases, Bak et al. reported that acute tears were consensually repaired, and the earlier the surgery was performed, the better the clinical outcomes were observed [7]. In contrast, chronic tears are more difficult to manage, regarding alteration of tissue quality and tendon retraction [17]. However, surgical repair seems to be the preferred option as excellent or good outcomes occur in more than 90% of operated patients, versus 17% of conservatively treated patients (best choice for elderly/sedentary patients or in muscle belly tears) [9, 11, 35].

Previous studies reported that patients with chronic PM tears managed with direct repair obtain similar clinical outcomes than acute repairs [6, 9, 12]. However, a graft is required when extensive surgical release of the PM belly muscle does not allow direct repair [15]. Fascia lata or Achilles tendon allografts are widely used for reconstruction of PM tendon [10, 19, 35, 36]. Allografts avoid donor-site morbidity and can be easily tailored to fill the gap. Drawbacks are disease transmission, delayed graft incorporation, and increased risk of retear [37]. Previous studies reported that sterilization with gamma irradiation could result in impaired biomechanical properties [38]. Thus, recent publications do not advocate irradiated tendon allograft for anterior cruciate ligament reconstruction [39, 40]. Sherman et al. demonstrated the high load to which PM tendon is exposed [23]. As for biomechanical properties, autograft seems therefore to be more adapted for PM reconstruction.

Dehler et al. reported a reconstruction technique with Human extracellular matrix scaffold device [15]. The use of dermal allograft has been successfully reported in rotator cuff augmentation [41], arthroscopic superior capsule reconstruction [42], and open revision repair [43] in patients with irreparable rotator cuff tears. This graft eliminates donor-site morbidity and the time to prepare the autograft and could have a better biologic incorporation than tendon allograft. However, studies reporting this technique have short clinical follow-up and indication being limited to rotator cuff repair. A graft thickness of 1 mm could be insufficient for PM tendon reconstruction.

For the tendon reconstruction and to fill the gap, ipsilateral hamstrings autograft was our graft choice. The advantages of this technique are (1) using autograft leads to both complete biocompatibility and safety regarding diseases transmission, (2) hamstring graft allows filling a significant gap, and (3) it is tailored to restore the anatomy of the PM tendon (fan-shape). The drawbacks are donor-site morbidity including injuries of the saphenous nerve [44]. A recent systematic review seems to suggest lower rate of neurological impairment adopting an oblique incision [45], which corresponded to our harvesting method.

The success of PM tendon reconstruction requires solid incorporation of the tendon graft within the bone tunnel to enable its histological remodeling. Numerous graft fixation devices are reported in the literature. To optimize graft incorporation, interference screw was our choice, with 2 cm autograft driven in the bone tunnel. This fixation technique was easy to perform, resulting in a solid fixation of the graft in tubular bone of the humerus, as described in subpectoral tenodesis of the long head of the biceps. Drawbacks using an interference screw could be the risk of humeral fracture [46, 47], screw migration, and cyst formation [48] as described with anterior cruciate ligament reconstruction. Furthermore, this tendon reconstruction was not anatomical, the native humeral insertion of the PM measuring near 5 cm. In our case, regarding the gap, there was no possibility of using a superior second screw to perform a more anatomical double bundle tendon reconstruction with hamstring autograft.

Pectoral Major tears are mainly described in young male weight lifters and in high-performance athletes. We recognize that this profile did not correspond entirely to our case, who did not practice sport. However, this young patient had to be considered as a heavy manual worker who had a high demand corresponding to his return to work. The excellent clinical outcomes at one-year follow-up suggest that PM tear with major tendon retraction can be reliably managed with hamstring autograft reconstruction, using an interference screw for fixation device. This technique has proven its simplicity and efficiency to fill the gap. Biomechanical studies, although already validated for subpectoral tenodesis, could be considered for this technique.
